# Free-breathing myocardial T2 measurements at 1.5T

**DOI:** 10.1186/1532-429X-13-S1-P11

**Published:** 2011-02-02

**Authors:** Maelene Lohezic, Anne Menini, Jean-Marie Escanyé, Pierre-Yves Marie, Damien Mandry, Pierre-André Vuissoz, Jacques Felblinger

**Affiliations:** 1 GE Healthcare / IADI Lab, Nancy, France; 2 INSERM U947, Nancy, France; 3 Nancy-Université, Nancy, France; 4 CHU Nancy, Nancy, France

## Introduction

Myocardial T2 mapping is a valuable tool for tissue characterization and oedema visualization. For instance, it is used to detect early rejection of heart transplant [1]. T2 values are usually estimated by performing several black blood Fast Spin Echo (FSE) sequences with different Echo Times (TE), what requires multiple breath holds. Successive apneas could lead to misregistration between images and to patient discomfort. A method allowing free breathing myocardial T2 measurements has been recently proposed and evaluated at 3T [2]. Results at 1.5T are presented here.

## Purpose

This study aims at demonstrating the feasibility of free-breathing myocardial T2 mapping at 1.5T.

## Methods

### MRI experiments

Five healthy volunteers underwent cardiac examination at 1.5T (SIGNA HDxt, GE Healthcare, Milwaukee, WI). Two sets of ten images with different TE were acquired with a conventional cardiac-gated black blood FSE sequence at mid-cavity short axis view, one during breath hold and the other one during free breathing. The same parameters were used, except for echo train length (ETL) (Table 1).ETL was set at 24 to keep the breath hold duration short, whereas 16 echoes were used for free-breathing acquisitions. Raw data from the free breathing acquisitions were recorded. Signals from a respiratory belt were carried by a custom Maglife patient monitoring system (Schiller Medical, France) and recorded with a dedicated home-made hardware [3].

**Table 1:** Acqusition parameters

TE	10 to 75 ms
TR	2RR
TI	500 ms
Matrix size	128X128
FOV	36 cm
BW	62.5kHz
Slice thickness	10mm
ETL	16 or 24

### Post-processing

First, the ten breath-held images were registered manually. Then, the T2 map was obtained using a mono-exponential model to fit the T2 signal versus the echo time decay curve, on a pixel-wise basis.

### Free breathing reconstruction

Using physiological signals extracted from the respiratory belt, the method presented in [2] was used to obtain an artefact-free proton density weighted image r0 and a T2 map from the free breathing raw data set. For the sake of comparison, six segments were drawn on the left ventricle myocardium. The mean value of each ROI was then used to get 6 myocardial T2 values.

## Results

**Figure 1:**
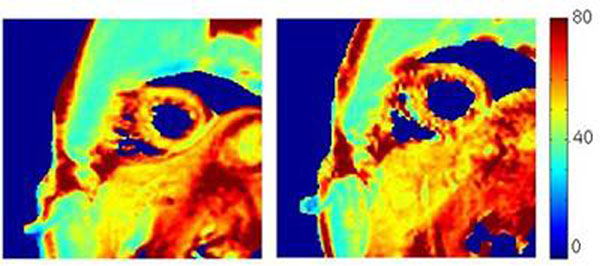
T2 maps obtained on one volunteer in breath hold (left) and in free breathing (right)

Like at 3T, there was no significant differences between the two sets of myocardial T2 values (paired Student T-test, p=0.17). The free breathing T2 maps were in good agreement with the breath-held ones and respiratory artefacts were widely reduced in r0 (Fig. 1).

## Conclusions

The proposed free breathing method allows performing accurate T2 mapping at 1.5T with no additional acquisition time.
